# Assessing the impact of COVID-19 on routine immunization in Sierra Leone

**DOI:** 10.1186/s12889-024-19221-2

**Published:** 2024-07-05

**Authors:** Umaru Sesay, Hector Mario Serna-Chavez, Gebrekrstos Negash Gebru, Jia Bainga Kangbai, Uzoma Ogbonna, James Sylvester Squire, Mirjam Irene Bakker

**Affiliations:** 1 KIT Royal Tropical Institute, Amsterdam, The Netherlands; 2 National Surveillance Program, Directorate of Health Security and Emergencies, Emergency Operation Center, National Public Health Agency, Wilkinso Road, Freetown City, Sierra Leone; 3 Sierra Leone Field Epidemiology Training Program, Emergency Operation Center, National Public Health Agency, Wilkinson Road, Freetown City, Sierra Leone; 4 Africa Field Epidemiology Network, Freetown City, Sierra Leone; 5 Department of Environmental Health, Njala University, Bo City, Sierra Leone; 6 Tulane University School of Public Health and Tropical Medicine, New Orleans, USA

**Keywords:** COVID-19, Pandemic, Routine immunization, Vaccination coverage rate, BCG vaccine, Bacillus calmette–guérin, Measles-Rubella, Pentavalent vaccine, Health Information system, Public health emergencies, Sierra leone

## Abstract

**Background:**

The COVID-19 pandemic had a profound impact on healthcare systems and services, including routine immunization (RI). To date, there is limited information on the effects of the COVID-19 pandemic on RI in West African countries such as Sierra Leone, which had already experienced public health emergencies that disrupted its healthcare system. Here, we describe the impact of the COVID-19 pandemic on the RI of key antigens in Sierra Leone.

**Methods:**

We used vaccination data from the District Health Information System for BCG, measles-rubella 1 and 2, and pentavalent 1 and 3 antigens. We compared 2019, 2020, 2021, and 2022 annual coverage rates for the selected antigens at the national and district levels. We used the Pearson chi-square test to assess the difference between annual coverage rates between 2019 and 2020, 2020–2021, and 2021–2022.

**Results:**

National coverage rates for all antigens declined in 2019–2020, notably measles-rubella 1 and pentavalent 3 (-5.4% and − 4.9%). Between 2020 and 2021, there was an overall increase in coverage (+ 0.2% to + 2.5%), except for measles-rubella 2 (-1.8%). Measles-rubella antigens rebounded in 2021–2022, while others decreased between − 0.5 and − 1.9% in coverage. Overall, all district-level coverage rates in 2022 were lower than those in 2019. Most districts decreased between 2019 and 2022, though a few had a continuous increase; some had an increase/recovery between 2020 and 2021; some districts had recovered 2019 levels by 2022.

**Conclusion:**

The COVID-19 pandemic impacted Sierra Leone’s national BCG, measles-rubella, and pentavalent antigen immunization, which were not fully restored in 2022. Most districts experienced notable coverage declines during the pandemic, though a few reached or surpassed 2019 rates in 2022. Examining pandemic impact can benefit from a focus beyond the national level to identify vulnerable regions. Sierra Leone’s post-pandemic RI reestablishment needs targeted strategies and continual investments for equitable access and coverage, as well as to prevent vaccine-preventable diseases.

**Supplementary Information:**

The online version contains supplementary material available at 10.1186/s12889-024-19221-2.

## Background

Worldwide, the coronavirus disease (hereafter, COVID-19), an infectious disease caused by the Severe Acute Respiratory Syndrome Coronavirus 2 (SARS-CoV-2) [1], had a profound impact on healthcare systems and services, including routine immunization [2]. As of 11 February 2024, approximately 774,631,444 infections and 7,031,216 deaths from the coronavirus disease have been reported globally [3]. The pandemic disrupted reproductive, maternal, and child health interventions, and with lock-downs and traveling restrictions to curtail infections, routine immunization (RI) became especially challenging [2, 4]. Recent studies have evidenced the impact of COVID-19 pandemic on vaccination coverage in children under 24 months in countries like Italy, Canada, the United States of America [5–7], as well as in a few low and middle-income countries [4, 8–10]. In the latter, special attention is needed to avoid reversing the progress against vaccine-preventable diseases (VPDs) such as measles [11]. In the African continent, it was estimated that before the COVID-19 pandemic, one out of every five children did not receive the recommended childhood vaccinations [12]). Such pre-pandemic status reinforces the need to assess the impact of the pandemic and the measures instituted to curb infections on vaccination coverage.

Over the last three decades, Sierra Leone has experienced multiple public health emergencies in its history: a civil war (1991 to 2002) [13], an Ebola outbreak (2014 to 2015) [14], severe flooding (2017) [15], and, most recently, the COVID-19 pandemic [16]. It is reported that the previous emergencies in Sierra Leone led to a drop in fully immunized children, from 68% in 2013 to 56% in 2019 [17]. During the COVID-19 pandemic, the Government of Sierra Leone activated the National Public Health Emergency Operation Centre Level 2 to prevent the entrance of the SARS-CoV-2 virus [18]. Despite this multi-sectorial response, Sierra Leone detected its first COVID-19 case on 31 March 2020 [16]. In countries like Sierra Leone, the impact of the COVID-19 pandemic on RI could have compounded effects as they were working on rebuilding their health systems and infrastructure after previous public health emergencies. To date, the extent of the impact of the pandemic on RI in Sierra Leone remains unquantified. Addressing this knowledge gap is key to informing stakeholders on developing evidence-based strategies and policies for RI protocols during public health emergencies, re-establishing RI efforts based on coverage gaps, and assessing the risk of VPDs. In this study, our goal is to assess the COVID-19 effect on the coverage of key vaccines in Sierra Leone. We analyze this impact on vaccination coverage at both national and district levels, focusing on the Bacillus Calmette–Guérin (BCG), measles-rubella 1 and 2, and pentavalent 1 and 3 antigens.

## Methods

### Design and study setting

We used data from the District Health Information Software 2 (DHIS2) to retrieve vaccination data at the national and district levels in Sierra Leone. For this study, we considered the BCG, measles-rubella, and pentavalent antigens because they are used as indicators to determine whether children have acquired full immunity [19]. Moreover, these antigens serve as a proxy for the other antigens that are simultaneously administered to the same target population [20].

We decided to focus on analyzing changes in coverage rates for the years 2019, 2020, 2021, and 2022 because these covered the acute period of COVID-19 infections as well as a “post-pandemic” period. This time-span would allow us to compare the change in coverage rates during the peak of the pandemic with those afterward. We performed comparisons using the Pearson chi-square test for two-year time intervals: 2019–2020, 2020–2021, and 2021–2022. This comparison of two-year time intervals was performed for annual coverage rates at the national level as well as for each of the 16 districts in Sierra Leone. Due to the order of magnitude in the number of doses and the target population, even small differences in coverage rates of antigens will likely result in statistical significance. To avoid this bias using p-values to assess the relevance of results, we focus on presenting and discussing the effect sizes instead.

### Data source

The source of routine immunization data was the District Health Information Software 2 (DHIS2) [21]. The DHIS2 is a global system used to manage health data, with reports at the facility, district, and national level [22]. It is the data repository used by all health facilities in Sierra Leone. Its record for Sierra Leone goes back to 2012 [23]. At the health facility level, routine immunization data of children younger than twelve months old are captured individually and recorded in the facility’s register. Monthly, the immunization data of each facility are aggregated and entered into DHIS2 by a monitoring and evaluation officer. For Sierra Leone, the DHIS2 is the most comprehensive source of RI data and it is used by clinicians, public health professionals, and academics.

### Data extraction

We extracted from the DHIS2 the annual total number of doses administered for BCG, measles-rubella 1 and 2, and pentavalent 1 and 3 for 2019, 2020, 2021, and 2022 for each district in Sierra Leone [21]). To extract the data from the DHIS2, we selected the “immunization” element. These data represent the vaccination doses. We then selected the key antigens (BCG, measles-rubella 1 and 2, pentavalent 1 and 3) and specified the year of interest by selecting every month in 2019, 2020, 2021, and 2022. After selecting the period, we designated the “organizational units.” This specifies the aggregation level of the data: national or district-level data, in our case. Once we selected these features in the DHIS2 system, we set organizational units (national, then districts) as rows and years as columns. The data was downloaded in Microsoft Excel format. After downloading the DHIS2 vaccination data, we merged it with the projected population data for each year.

We extracted the size of the target population for each focus year in this study from the 2015 population base census projection [24]. We extracted the live birth population from the 2015 population projection at the district and national levels to calculate coverage for BCG, and the surviving infant population projection to calculate the coverages for measles-rubella 1 and 2, and pentavalent 1 and 3 coverage. In Sierra Leone, the live birth population accounts for 4% of the total population, and the surviving infants account for 3.7% of the total population per each year [20].

### Data Management

Data files used in this study did not contain any private information such as patient or facility names. The raw files as well as the complete dataset used for our analyses were kept in MS Excel format and protected by password in a secure cloud storage system. Only authors conducting the data analyses and developing visualizations were granted access to the complete dataset.

### Operational definition

Coverage rate is defined as the proportion of children who received the prescribed dose of a vaccine per specific target population [19].

Live birth (BCG target population) is defined as the child alive extracted from its mother, regardless of the duration of the pregnancy [25].

Surviving infants (measles-rubella and pentavalent target population) are defined as children who survive their first year after birth [26].

### Data analysis

The annual coverage rate was computed as the proportion of the total number of doses administered per specific antigen divided by the target population per year [19]. This was calculated for each of the focus antigens at the national level as well as for each of the 16 districts in Sierra Leone.

For the national-level analysis of changes in annual coverage rates of BCG, measles-rubella 1 and 2, and pentavalent 1 and 3, we compared 2019 to 2020 rates; 2020 to 2021; and 2021 and 2022. We used Pearson’s chi-square test to assess whether the differences in annual coverage rates were independent of each other using the R-software, version 4.2.0 [27]. Because the size of the target population and the number of vaccine doses administered nationally are large, all differences in the chi-square test will likely be significant at the third level (*p* < 0.001). Therefore, we focused our discussion on the changes in annual coverage rates between the focus years. We present changes in coverage rates at national dumbbell plots developed in R v4.2.0 [27]. A table with the data on doses, target population, coverage rates, and the outcome of comparing rates between years using Pearson’s chi-square test is available in Additional Information (Supplementary Table 1).

To assess the change in BCG, measles-rubella 1 and 2, and pentavalent 1 and 3 immunization at the district level, we first calculated the annual coverage rates for 2019, 2020, 2021, and 2022 for each district. This was done by dividing the number of doses in that district (obtained from the DHIS2) by the (projected) target population in that year. In some instances, this led to a district-level annual coverage rate > 100% because the number of doses administered was greater than the target population projected for that district, for that year. This is a known artifact when working with projections of population data. We did not cap or correct coverages > 100% estimates as done in other studies [28]. We consider these districts with likely a high level of vaccination coverage, but not fully vaccinated.

At the district level, we focused on analysing the trends in coverage rates between 2019 and 2022. This allowed us to determine if coverage of any antigen in any district has changed and whether it recovered to 2019 levels in 2022. We also conducted the two-year interval comparison using Pearson’s chi-square test for each antigen, for each district. In the main body of this article, we present time series matrixes for BCG, measles-rubella 1, and pentavalent 1 coverage rates at the district level, developed in R v4.2.0 [27]. For all antigens, tables with the district-level data on doses, target population, coverage rates, and the outcome of comparing rates between years are available in Additional Information (Supplementary Tables 2–6).

### Data validation

We explored different sources to validate the immunization data used for this study. The global databases available, namely the SLDHS 2019 (17) and the WUENIC database [29], were not deemed suitable for data validation. The information available in SLDHS 2019 is limited to 2018, prior to the focus years of this study. The estimates of the WUENIC database for Sierra Leone only cover the BCG antigen and are not fully independent because they are based on reported data. The reported data in WUENIC is the same as the one uploaded to the DHIS2. The survey data available in WUENIC only covered the BCG antigen for the year 2019 at the national level. This is not sufficient for data validation.

At the district level, we found a recent study with estimates on vaccination coverage for our key antigens [30]. They studied the immunization of children aged 10–23 months in the districts of Bombali, Tonkolili, and Port Loko in Sierra Leone. We intended to do a partial data validation of our findings using the coverages reported in that study. However, we could not do the data validation as the study reported metrics of fully immunized children only while this study reports antigens-specific coverage rates.

## Results

### Changes in vaccination coverage at the national level

**Fig. 1:**
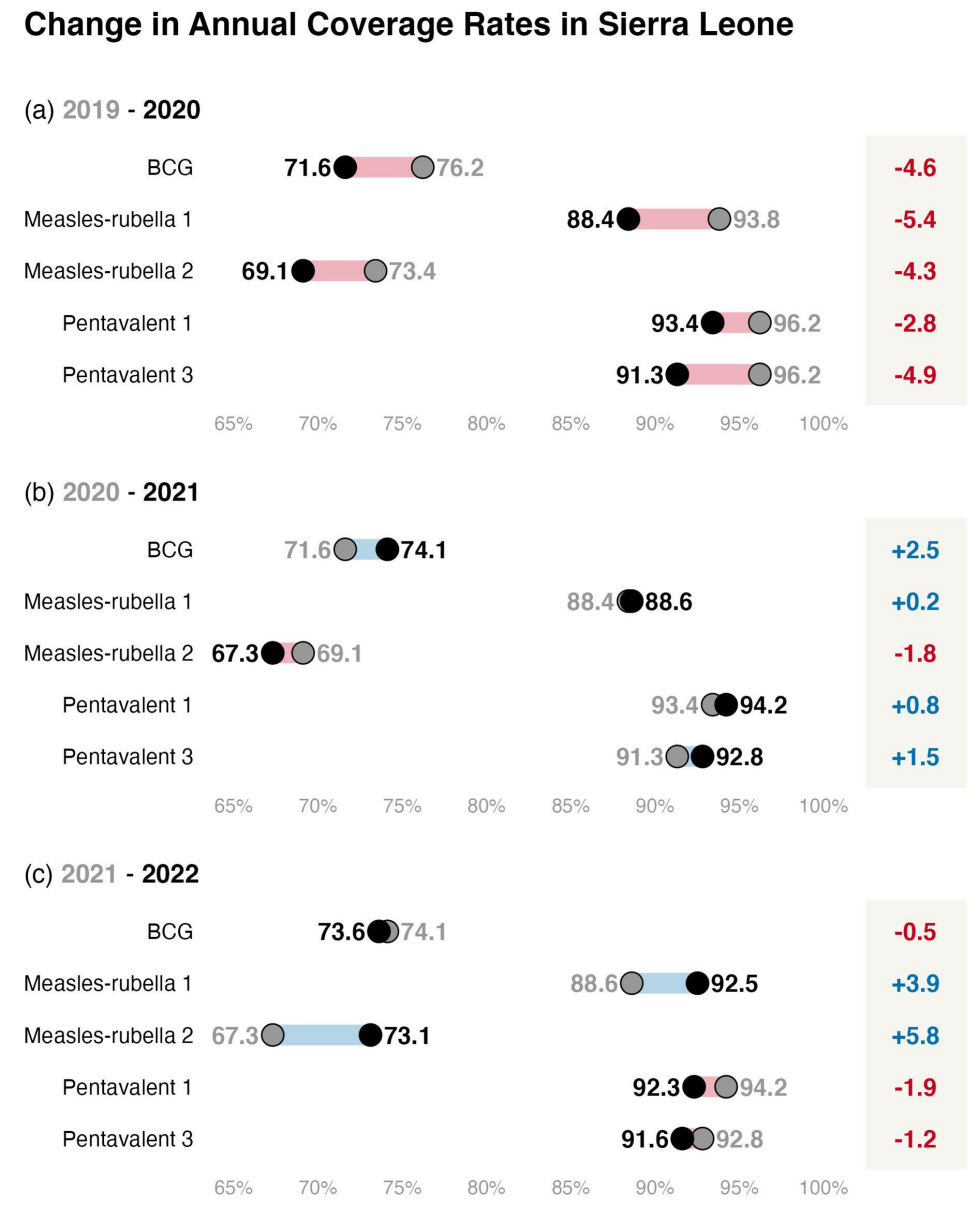
Changes in annual coverage rates at the national level. Each panel presents the change comparison between the focus years: (**a**) 2019–2020, (**b**) 2020–2021, and (**c**) 2021–2022. The difference between each comparison is presented on the right-hand side of the plot. Red lines and values represent a decrease in annual coverage; blue means an increase. All values are in percentage points of the annual coverage rate (%)

Between 2019 and 2020, BCG, measles-rubella 1 and 2, and pentavalent 1 and 3 showed a decrease in coverage rate (Fig. 1a). Measles-rubella 1 showed the greatest decrease (-5.4%) followed by pentavalent 3 (-4.9%) and BCG vaccine (-4.6%). Between 2020 and 2021, all antigens showed a moderate increase in annual coverage rate, except measles-rubella 2 (Fig. 1b). Measles-rubella 2 decreased coverage by -1.8%. BCG exhibited the greatest increase in annual coverage with + 2.5% in 2021, compared to 2020. The coverage of pentavalent 3 increased by + 1.5%. Measles-rubella 1 and pentavalent 1 increased coverage by < 1%. Between 2021 and 2022, only the measles-rubella antigens showed an increase in coverage (+ 3.9% and + 5.8%, for measles-rubella 1 and 2, respectively, Fig. 1c). Pentavalent 1 and 2 antigens had a decrease of -1.9 and − 1.2% in 2022, respectively, compared to 2021 levels. The BCG had a slight decrease of -0.5%.

None of the antigens showed a consistent decrease or increase in annual coverage throughout the four years (2019 to 2022, Fig. 1). Only the measles-rubella 1 antigen shows an increase in annual coverage since 2020. Even so, the national annual coverage for measles-rubella 1 in 2022 was lower than that recorded in 2019 (92.5% and 93.8%, respectively). For BCG, measles-rubella 2, and both pentavalent antigens there was no consistent recovery in annual coverage since 2020. For all antigens, the annual coverages in 2022 were lower than those in 2019. Overall, BCG and measles-rubella 2 show a relatively low coverage since 2019 (between 76 and 73%, Fig. 1).

### Change in annual coverage rate in Sierra Leone’s districts

**Fig. 2:**
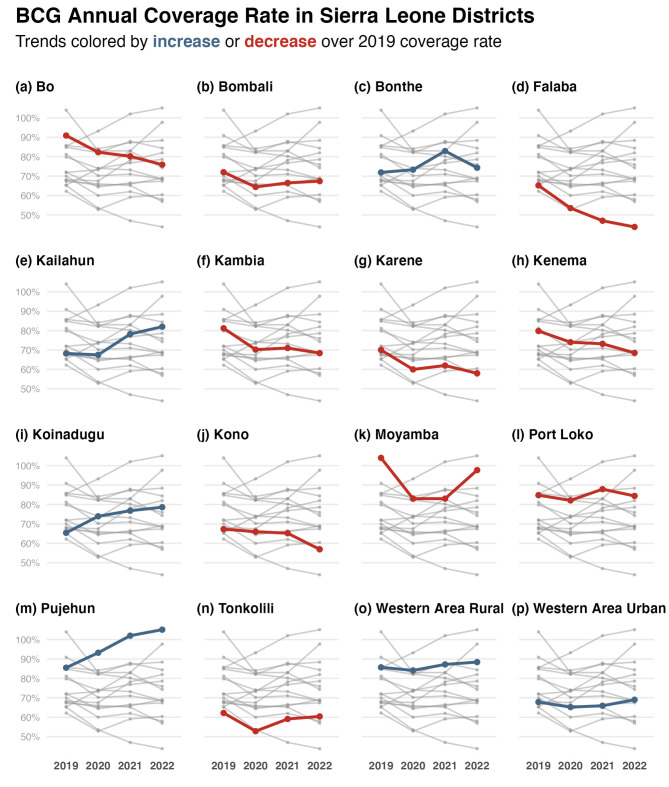
BCG mean annual coverage rates between 2019 and 2022 in Sierra Leone’s districts. Each panel presents the mean annual coverage rates of all districts: the focus district is presented in colour, and all others in grey. Red points and lines sign signify that the 2022 coverage rate is below the 2019 value; blue is the opposite. Some coverages exceeded 100% because the vaccination doses administered exceeded the target population projected for that district for that year

For BCG, the majority of districts in Sierra Leone showed a decrease in annual coverage rate from 2019 to 2022 (Fig. 2). Six districts (Bo, Falaba, Kambia, Karene, Kenema, and Kono) showed a consistent, sustained decrease in BCG coverage between 2019 and 2022. The majority of districts had a decrease in BCG coverage between 2019 and 2020. Only three districts showed an increase in BCG coverage in 2019–2020: Bonthe, Koinadugu, and Pujehun. For the rest, this period resulted in a decrease in coverage. Though the overall pattern showed a decrease in annual coverage, Moyamba and Tonkolili showed some increase in coverage between 2020 and 2022 (Fig. 2k, n). Only six of the 16 districts had an increase in BCG annual coverage that resulted in 2022 values being greater than those in 2019 (Supplementary Table 2). These districts are Bonthe, Kailahun, Koinadugu, Pujehun, and the Western Area Rural and Urban (Fig. 2, panels with blue lines).

**Fig. 3:**
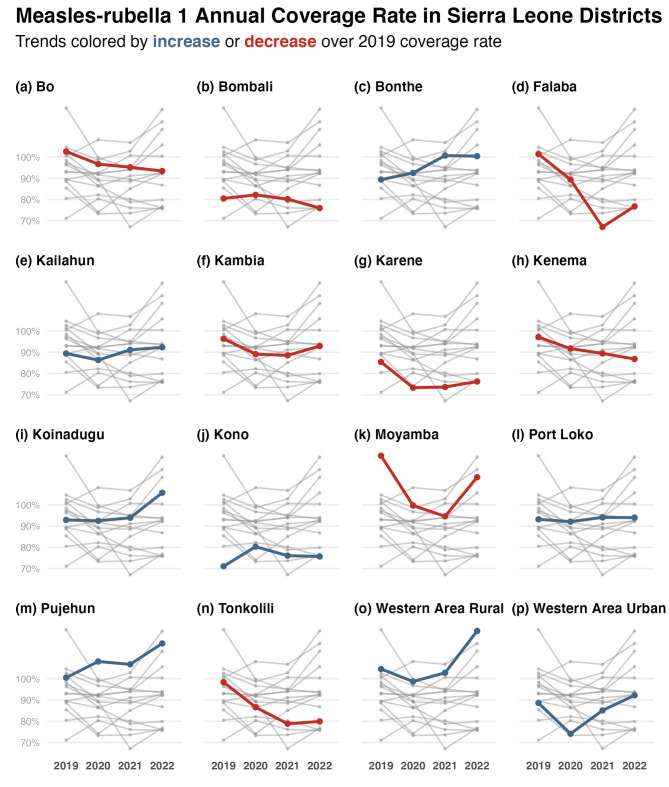
Measles-rubella 1 mean annual coverage rates between 2019 and 2022 in Sierra Leone’s districts. Each panel presents the mean annual coverage rates of all districts: the focus district is presented in colour, and all others in grey. Red points and lines signify that the 2022 coverage rate is below the 2019 value; blue is the opposite. Some coverages exceeded 100% because the vaccination doses administered exceeded the target population projected for that district for that year

The majority of districts in Sierra Leone showed a decrease in vaccination coverage for measles-rubella 1 between 2019 and 2020 (Fig. 3). Seven districts (Bo, Bombali, Falaba, Kambia, Karene, Kenema, and Tonkolili) showed a consistent, sustained decrease in measles-rubella 1 coverage between 2019 and 2022. Four districts, including Bombali, Bonthe, Kono, and Pujehun, had increased coverage between 2019 and 2020. During the COVID-19 pandemic era (2020 and 2022), almost half of all districts in Sierra Leone (Bonthe, Kailahun, Karene, Koinadugu, Port Loko, Western Area Rural, and Western Area Urban) showed a sustained increase (Fig. 3, panels with blue lines). Half of the Sierra Leone districts reached 2019 coverage rates in 2022, (namely Bonthe, Kailahun, Koinadugu, Kono, Port Loko, Pujehun, Western Area Rural and Urban) (Supplementary Table 3).

**Fig. 4:**
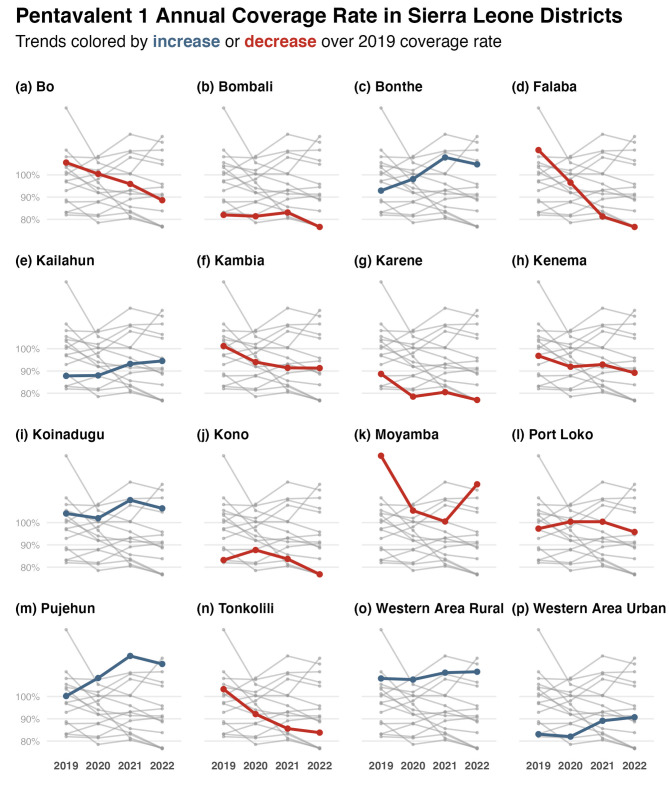
Pentavalent 1 mean annual coverage rates between 2019 and 2022 in Sierra Leone’s districts. Each panel presents the mean annual coverage rates of all districts: the focus district is presented in colour, and all others in grey. Red points and lines signify that the 2022 coverage rate is below the 2019 value; blue is the opposite. Some coverages exceeded 100% because the vaccination doses administered exceeded the target population projected for that district for that year

Regarding Pentavalent 1, the majority of the districts showed a decrease in vaccination coverage between 2019 and 2020 (Fig. 4). Within the same period, five districts (Bonthe, Kailahun, Kono, Port Loko, and Pujehun) showed an increase in annual pentavalent 1 coverage. During the COVID-19 pandemic era (2020 and 2022), six districts (Bo, Falaba, Kambia, Karene, Kenema, and Tonkolili) showed a consistent, sustained decrease in pentavalent 1 coverage. While four of the districts (Kailahun, Pujehun, Western Area Rural, and Western Area Urban) show an increase in pentavalent 1 coverage. Only six districts reached 2019 coverage in 2022 (namely, Bonthe, Kailahun, Koinadugu, and Pujehun) (Fig. 4, panels with blue lines).

Between 2019 and 2022, there seemed to be no consistent geographical pattern in the changes in coverage rates for any of the BCG, measles-rubella 1, and pentavalent 1 antigens. Districts in provinces further away from the capital did not consistently show decreases in annual coverages of the focus antigens. What is evident in the district time series is how all but two districts show consistent decrease or increase in annual coverage for BCG, measles-rubella 1, and pentavalent 1 antigen between 2019 and 2022. Kono and Port Loko districts, both in different provinces of Sierra Leone, show a 2022 coverage rate of measles-rubella 1 greater or equal to that in 2019 (Figs. 2 and 3, and 4, panels j and l). For all other districts, the patterns on whether they recovered the 2019 annual coverage levels by 2020 were conserved for BCG, measles-rubella 1 and 2, and pentavalent 1 and 3 (Supplementary Tables 2,3,4,5, and 6).

## Discussion

This study shows that the annual coverage for BCG, measles-rubella 1 and 2, and pentavalent 1 and 3 antigens declined as expected during COVID-19 pandemic and how, at the national level, these coverage rates had not been recovered by the end of 2022. Consistent with the national pattern, such decreases were also evident for most districts in Sierra Leone. For the majority of districts in Sierra Leone, the 2019 annual coverage rates of BCG, measles-rubella 1, and pentavalent 1 were not recovered by 2022. There was no evident geographical pattern in the changes in annual coverage at the district level regarding provinces.

The decrease in annual coverage rates between 2019 and 2020 were as expected. During the peak period of the COVID-19 pandemic, the Government of Sierra Leone diverted resources and implemented several control strategies to halt the spread of the COVID-19 virus [30]. The implementation of these strategies disrupted the operation of RI services, likely leading to the observed decrease in vaccination coverage [30]. Other studies in low- and middle-income countries in Africa have also shown such a decrease during the peak of the pandemic. For example, studies in Liberia [31] reported a decreased vaccination coverage of BCG, pentavalent, and measles antigens in the first six months of 2020 compared to those in 2019. In Guinea [32], a study showed a drop from 95 to 77% median monthly coverage in the first months of 2020. The same drop is evident in countries in the global north [5–7].

This study also found that, between 2020 and 2021, all antigens recorded an increase in annual coverage, except measles-rubella 2. On March 8, 2021, the Government of Sierra Leone launched the COVID-19 vaccinations [33]. During the COVID-19 vaccination, healthcare workers were tasked to integrate RI service in the COVID-19 vaccination, and political leaders were tasked to dispel negative rumours about COVID-19 and RI uptake, among others [34]. The implementation of these measures may have contributed to the increase in annual RI coverage. A similar pattern was found in a study conducted in Gambia, where, after the changes in coverage rates during the first and second wave of the pandemic, there was an increase in Hep0 and pentavalent 1 coverage before and after the third wave in 2021 [35]. The authors reported that RI services were integrated into the COVID-19 vaccination campaign, likely contributing to the increase in coverage during the pandemic [35]. Both Sierra Leone and the Gambia share similar geographical features and healthcare system operations.

At the national level, by 2022 none of the focus antigens had reached the annual coverage levels in 2019. Even though Sierra Leone implemented several interventions to improve RI uptake [34], the already existing determinant factors including poverty (56.7% of the population lived below $1.25 a day) [36] and low education (more than 80% illiterate) [37] worsened by the COVID-19 pandemic contributed to the low RI coverage in 2022 compared to 2019. Because the annual coverage in 2022 did not recover in 2019, and the coverages were also below the EPI target (≥ 95%) (20), it is likely that the majority of children were vulnerable to VPD even before the pandemic, and their risk of infection further increased during and after the pandemic. This finding is different from a study conducted on the status of Routine Immunization in The World Health Organization African Region Three Years into the COVID-19 Pandemic [38]. In that study, the authors reported MCV2 and HEPBB assessed at the national level recovered 2019 coverage in 2022.

Examining pandemic impact can benefit from a focus beyond the national level to identify vulnerable regions. Most districts in Sierra Leone had a consistent decrease in coverage rate between 2019 and 2022, though a few show an increase. And the districts with the greatest changes in coverage rates were not necessarily those farthest away from Freetown. For instance, the Western Area Urban District, which hosts the RI vaccination distribution center and recorded the first COVID-19 case, had a greater decrease in vaccination coverage compared with some districts located far away from the vaccination center, such as Pujehun district. To our knowledge, there is no straightforward explanation for the heterogeneity in vaccination coverage across districts in Sierra Leone. A qualitative or mixed-methods study across districts can help understand the reasons for the heterogeneity in RI vaccination coverage during the COVID-19 pandemic.

There seems to be some consistency between the districts in Sierra Leone that did not recover 2019 coverage levels of BCG, measles-rubella 1, and pentavalent 1 by 2022. Because the antigens are part of the same vaccination schedule plan [20], the challenges imposed by the pandemic coupled with the control measures instituted to stop the pandemic affected the uptake of RI vaccines equally. This finding is similar to a study conducted in Burkina Faso [39], where the authors reported consistency for RI vaccine coverage, except for newly introduced ones. Burkina Faso and Sierra Leone share similar geographical features.

### Limitation of study

We acknowledge a few limitations to the study. First, the uncertainty around the estimated size of the target population is used to calculate the annual coverage rate. We followed a standard protocol and used the projected population estimates derived from the updated 2015 national population census held in Sierra Leone [24]. Bias in these projections as well as not accounting for changes in the target population during the pandemic might have influenced our findings, leading to an overestimation or underestimation of annual coverage rates at the national and district level.

Second, we relied on the reported DHIS2 data to calculate coverage rates for the different antigens. The limited resources to routinely monitor the number of doses of data entered into the system and to perform comprehensive quality checks at the health facility level could mean some inaccuracies in vaccination data. It is recognized that, because immunization coverage is measured on target, some health facilities could likely have overreported or underreported the number of doses given to meet their routine immunization target. Nonetheless, in Sierra Leone data entry errors are routinely picked up during the quarterly (by national EPI officers) and monthly (by district officers) checks. Though we cannot discard noise in the vaccination data, we trust our estimates are robust.

Finally, we focused on the changes in annual coverage rates for key antigens. The nature of the patterns we uncovered at national and district level will likely have different causes. Elucidating them would require a different multivariate analysis to characterize the effect of different factors on changes in coverage rates during the COVID-19 pandemic.

### Implication of study findings

The decrease in annual coverage at the district and national level suggests a potential increased VPD outbreaks [5, 9, 30, 40] if swift measures are not enacted to improve the coverage rate. Because Sierra Leone is challenged in rebuilding its healthcare system, the COVID-19 pandemic disruption on RI uptake has reversed the progress made in improving RI vaccine uptake. At the individual level, the indirect costs such as transportation will increase tremendously, thereby affecting RI service utilization [41]. At the community level, stakeholders will be tasked with devising strategies such as community by-laws to fight against future pandemics or outbreaks. The implementation of these strategies will potentially affect the supply and demand of RI services.

To enhance RI uptake during future public health emergencies, the Ministry of Health should provide an adequate stock of RI vaccines, provide incentives to healthcare workers for outreach services, and supplemental and mop-up campaigns should be implemented promptly and RI intensify. Additionally, stakeholders should train healthcare workers on responding to future public health emergencies, and use phone calls or text messages to remind caregivers of vaccination schedules [6]. In the medium or long term, stakeholders should develop pandemic and response protocols; and develop a platform to utilize private sector expertise and resources for policy development and implementation. Also, stakeholders should develop electronic medical records to track defaulter children; and embed a performance-based system that is anchored on problem-solving, innovation, and transparency. Finally, set out a framework to understand the needs and priorities during the pre-post crisis phases [6, 34, 42].

## Conclusion

This study shows a decreased coverage for BCG, measles-rubella 1 and 2, and pentavalent 1 and 3 during the COVID-19 pandemic, with an incomplete recovery in 2022 at the national and district levels. Regardless of when Sierra Leone reported its first COVID-19 case, post-pandemic RI establishment needs targeted and specific policies and mechanisms to support sustaining RI services, and continual investment for equitable access to vaccines as well as to prevent VPDs during future public health emergencies.

### Electronic supplementary material

Below is the link to the electronic supplementary material.

Supplementary Material 1

## Data Availability

The datasets generated during the current study are available as supplementary files submitted with this manuscript.

## Abbreviations

COVID-19: Corona Virus Disease 2019
SARS-CoV-2: Severe acute respiratory syndrome coronavirus type two
RI: Routine Immunization
RMNCH: Reproductive, Maternal, and Child Health
VPD: Vaccine preventable disease
DHIS 2: District Health Information Software 2
