# Supplementary material for: Assessing the impact of COVID-19 on routine immunization in Sierra Leone
Source: BMC Public Health. 2024 Jul 5;24:1795. doi: 10.1186/s12889-024-19221-2 (PMC11225177; doi:10.1186/s12889-024-19221-2)
Supplement: Supplementary file 1 — Supplementary Material 1 [file 12889_2024_19221_MOESM1_ESM.docx]

Additional information

**Supplementary Table 1. Annual coverage rates of key antigens in Sierra Leone.**

| **Antigen** | **Annual Coverage Rate*** | | | |  |  |  |
| --- | --- | --- | --- | --- | --- | --- | --- |
|  | **2019** | **2020** | **2021** | **2022** | **Δ% 2019-2020** | **Δ%  2021-2020** | **Δ% 2022-2021** |
| **BCG** | 76.2 (240702/316058) | 71.6 (231866/324013) | 74.1 (245861/331915) | 73.6 (249846/339770) | -4.6 | 2.5 | -0.5 |
| **Measles-rubella 1** | 93.8 (266684/284452) | 88.4 (257647/291611) | 88.6 (272059/307022) | 92.5 (290766/314288) | -5.4 | 0.2 | 3.9 |
| **Measles-rubella 2** | 73.4 (208720/284452) | 69.1 (201439/291611) | 67.3 (206553/307022) | 73.1 (229663/314288) | -4.3 | -1.8 | 5.8 |
| **Pentavalent 1** | 96.2 (273728/284452) | 93.4 (272223/291611) | 94.2 (289263/307022) | 92.3 (290052/314288) | -2.8 | 0.8 | -1.9 |
| **Pentavalent 3** | 96.2 (273648/284452) | 91.3 (266248/291611) | 92.8 (285064/307022) | 91.6 (287782/314288) | -4.9 | 1.5 | -1.2 |

*Δ%: Delta in coverage rates.
*: Mean annual coverage calculated as the product of dividing the total number of routine immunization doses administered by the target population per specific year.*

**Supplementary Table 2.** **Annual coverage rates of BCG antigen in Sierra Leone’s districts.**

|  | **BCG Annual Coverage Rate*** | | | | **Δ% 2019-2020** | **Δ%  2021-2020** | **Δ% 2022-2021** |
| --- | --- | --- | --- | --- | --- | --- | --- |
| **Districts** | **2019** | **2020** | **2021** | **2022** |  |  |  |
| Bo | 90.9 (23309/25646) | 82.3 (22490/27237) | 80.2 (22457/27994) | 75.9 (20920/27570) | -8.6 | -2.1 | -4.3 |
| Bombali | 72.0 (13580/18849) | 64.4 (12450/19323) | 66.5 (13165/19795) | 67.4 (13664/20263) | -7.6 | 2.1 | 0.9 |
| Bonthe | 71.9 (6434/8948) | 73.3 (6563/8958) | 82.9 (7605/9176) | 74.3  (7146/9619) | 1.4 | 9.6 | -8.6 |
| Falaba | 65.2 (5965/9152) | 53.5 (5017/9382) | 47.0 (4520/9611) | 43.8  (4308/9838) | -11.7 | -6.5 | -3.2 |
| Kailahun | 68.2 (16004/23458) | 67.5 (16595/24572) | 78.2 (19672/25171) | 82.0 (20699/25218) | -0.7 | 10.7 | 3.8 |
| Kambia | 81.2 (12507/15396) | 70.2 (11047/15729) | 71.0 (11440/16113) | 68.4 (11316/16551) | -11 | 0.8 | -2.6 |
| Karene | 70.3 (8946/12725) | 60.2 (7850/13046) | 62.0 (8287/13364) | 58.3  (7978/13680) | -10.1 | 1.8 | -3.7 |
| Kenema | 79.8 (21680/27180) | 74.0 (20271/27409) | 73.2 (20543/28077) | 68.5 (20001/29219) | -5.8 | -0.8 | -4.7 |
| Koinadugu | 65.4 (5949/9092) | 73.9 (6888/9321) | 76.8 (7336/9548) | 78.6  (7681/9774) | 8.5 | 2.9 | 1.6 |
| Kono | 67.3 (15177/22554) | 66.0 (14352/21733) | 65.2 (14514/22264) | 56.9 (13797/24246) | -1.3 | -0.8 | -8.3 |
| Moyamba | 104.0 (14771/14198) | 83.0 (14071/16953) | 83.0 (14415/17366) | 97.7 (14908/15263) | -21 | 0 | 14.7 |
| Port Loko | 84.8 (20055/23658) | 82.1 (19924/24253) | 87.9 (21838/24845) | 84.4 (21474/25433) | -2.7 | 5.8 | -3.5 |
| Pujehun | 85.5 (13199/15440) | 93.2 (14748/15826) | 102.0 (16538/16212) | 105.1 (17438/16598) | 7.7 | 8.8 | 3.1 |
| Tonkolili | 62.2 (14244/22906) | 52.8 (12986/24596) | 59.1 (14879/25196) | 60.4 (14879/24624) | -9.4 | 6.3 | 1.3 |
| Western Area Rural | 85.7 (16972/19799) | 84.1 (17192/20446) | 87.2 (18262/20944) | 88.4 (18819/21284) | -1.6 | 3.1 | 1.2 |
| Western Area Urban | 67.8 (31910/47059) | 65.2 (29422/45139) | 65.9 (30490/46240) | 69.0 (34898/50589) | -2.6 | 0.7 | 3.1 |

*Δ%: Delta in coverage rates.
*: Mean annual coverage calculated as the product of dividing the total number of routine immunization doses administered by target population per specific year.
Note: Some coverage rates are >100% because the number of doses administered exceeded the target population.*

**Supplementary Table 3. Annual coverage rates of Measles-rubella 1 in Sierra Leone’s districts.**

| **Districts** | **Measles-rubella 1 Annual Coverage Rate*** | | | | **Δ% 2019-2020** | **Δ%  2021-2020** | **Δ% 2022-2021** |
| --- | --- | --- | --- | --- | --- | --- | --- |
|  | **2019** | **2020** | **2021** | **2022** |  |  |  |
| Bo | 102.6 (23682/23081) | 96.7 (23775/24594) | 95.2 (24659/25894) | 93.4 (23819/25502) | -5.6 | -1.5 | -1.8 |
| Bombali | 80.5 (13659/16964) | 82.2 (14297/17391) | 80.2 (14690/18310) | 76.0 (14245/18744) | 1.7 | -2 | -4.2 |
| Bonthe | 89.4 (7201/8053) | 92.5 (7458/8062) | 100.7 (8549/8488) | 100.4 (8937/8898) | 3.1 | 8.2 | -0.3 |
| Falaba | 101.4 (8353/8236) | 89.4 (7552/8444) | 67.1 (5967/8890) | 76.8 (6992/9100) | -12.0 | -22.3 | 9.7 |
| Kailahun | 89.4 (18879/21112) | 86.3 (19106/22115) | 91.1 (21214/23283) | 92.3 (21528/23327) | -3.1 | 4.8 | 1.2 |
| Kambia | 96.3 (13348/13856) | 89.1 (12617/14156) | 88.5 (13188/14904) | 92.9 (14219/15310) | -7.2 | -0.6 | 4.4 |
| Karene | 85.4 (9784/11453) | 73.3 (8609/11741) | 73.6 (9098/12362) | 76.2 (9642/12654) | -12.1 | 0.3 | 2.6 |
| Kenema | 97.1 (23755/24462) | 91.7 (22622/24668) | 89.4 (23219/25972) | 86.8 (23453/27027) | -5.4 | -2.3 | -2.6 |
| Koinadugu | 92.9 (7598/8183) | 92.5 (7758/8389) | 93.9 (8291/8832) | 105.7 (9560/9041) | -0.4 | 1.4 | 11.8 |
| Kono | 71.1 (14429/20299) | 80.3 (15700/19560) | 76.1 (15677/20594) | 75.7 (16981/22428) | 9.2 | -4.2 | -0.4 |
| Moyamba | 123.1 (15730/12778) | 99.7 (15208/15257) | 94.6 (15189/16064) | 113.0 (15951/14118) | -23.4 | -5.1 | 18.4 |
| Port Loko | 93.2 (19843/21292) | 92.1 (20103/21828) | 94.1 (21616/22981) | 94.0 (22116/23525) | -1.1 | 2 | -0.1 |
| Pujehun | 100.5 (13972/13896) | 108.1 (15403/14243) | 106.8 (16010/14996) | 116.6 (17894/15354) | 7.6 | -1.3 | 9.8 |
| Tonkolili | 98.4 (20290/20615) | 86.6 (19166/22137) | 78.8 (18359/23307) | 79.9 (18188/22777) | -11.8 | -7.8 | 1.1 |
| Western Area Rural | 104.6 (18634/17819) | 98.7 (18157/18401) | 102.8 (19914/19374) | 122.5 (24108/19688) | -5.9 | 4.1 | 19.7 |
| Western Area Urban | 88.6 (37527/42353) | 74.1 (30116/40626) | 85.1 (36419/42772) | 92.2 (43133/46795) | -14.5 | 11 | 7.1 |

*Δ%: Delta in coverage rates.
*: Mean annual coverage calculated as the product of dividing the total number of routine immunization doses administered by the target population per specific year.
Note: Some coverage rates are >100% because the number of doses administered exceeded the target population***.**

**Supplementary Table 4. Annual coverage rates of Measles-rubella 2 in Sierra Leone’s districts.**

| **Districts** | **Measles-rubella 2 Annual Coverage Rate*** | | | | **Δ% 2019-2020** | **Δ%  2021-2020** | **Δ% 2022-2021** |
| --- | --- | --- | --- | --- | --- | --- | --- |
|  | **2019** | **2020** | **2021** | **2022** |  |  |  |
| Bo | 75.0 (17313/23081) | 74.9 (18417/24594) | 75.2 (19471/25894) | 76.0 (19376/25502) | -0.10 | 0.3 | 0.8 |
| Bombali | 64.8 (10991/16964) | 66.3 (11526/17391) | 62.6 (11461/18310) | 61.5 (11527/18744) | 1.5 | -3.7 | -1.1 |
| Bonthe | 62.5 (5033/8053) | 59.2 (4773/8062) | 68.4 (5805/8488) | 77.4 (6889/8898) | -3.3 | 9.2 | 9 |
| Falaba | 76.7 (6316/8236) | 71.1 (6007/8444) | 43.3 (3846/8890) | 60.2 (5480/9100) | -5.6 | -27.8 | 16.9 |
| Kailahun | 86.1 (18186/21112) | 74.1 (16386/22115) | 74.9 (17439/23283) | 74.1 (17281/23327) | -12.0 | 0.8 | -0.8 |
| Kambia | 57.9 (8022/13856) | 60.5 (8561/14156) | 74.0 (11031/14904) | 82.4 (12611/15310) | 2.6 | 13.5 | 8.4 |
| Karene | 71.2 (8157/11453) | 63.7 (7479/11741) | 58.6 (7247/12362) | 64.6 (8170/12654) | -7.5 | -5.3 | 6 |
| Kenema | 78.9 (19297/24462) | 76.2 (18786/24668) | 70.9 (18405/25972) | 68.2 (18422/27027) | -2.7 | -5.3 | -2.7 |
| Koinadugu | 68.8 (5633/8183) | 69.3 (5816/8389) | 69.7 (6156/8832) | 88.1 (7968/9041) | 0.5 | 0.4 | 18.4 |
| Kono | 53.2 (10808/20299) | 64.1 (12545/19560) | 55.4 (11417/20594) | 63.7 (14295/22428) | 10.9 | -8.7 | 8.3 |
| Moyamba | 110.5 (14118/12778) | 91.0 (13882/15257) | 84.3 (13540/16064) | 96.2 (13588/14118) | -19.5 | -6.7 | 11.9 |
| Port Loko | 72.0 (15322/21292) | 76.0 (16591/21828) | 75.6 (17384/22981) | 87.8 (20650/23565) | 4.0 | -0.4 | 12.2 |
| Pujehun | 90.7 (12604/13896) | 93.7 (13341/14243) | 88.2 (13220/14996) | 92.4 (14183/15354) | 3.0 | -5.5 | 4.2 |
| Tonkolili | 78.7 (16225/20615) | 71.4 (15811/22137) | 54.2 (12641/23307) | 56.4 (12844/22777) | -7.3 | -17.2 | 2.2 |
| Western Area Rural | 73.1 (13030/17819) | 64.2 (11807/18401) | 66.5 (12885/19374) | 95.5 (18792/19688) | -8.9 | 2.3 | 29 |
| Western Area Urban | 65.3 (27665/42353) | 48.5 (19701/40626) | 57.5 (24605/42772) | 59 (27587/46795) | -16.8 | 9 | 1.5 |

*Δ%: Delta in coverage rates.
*: Mean annual coverage calculated as the product of dividing the total number of routine immunization doses administered by the target population per specific year.
Note: Some coverage rates are >100% because the number of doses administered exceeded the target population.*

**Supplementary Table 5.** **Annual coverage rates of Pentavalent 1 in Sierra Leone’s districts.**

|  | **Pentavalent 1 Mean Annual Coverage Rate*** | | | | **Δ% 2019-2020** | **Δ%  2021-2020** | **Δ% 2022-2021** |
| --- | --- | --- | --- | --- | --- | --- | --- |
| **Districts** | **2019** | **2020** | **2021** | **2022** |  |  |  |
| Bo | 105.5 (24360/23081) | 100.4 (24704/24594) | 95.9 (24844/25894) | 88.6 (22586/25502) | -5.1 | -4.5 | -7.3 |
| Bombali | 82.0 (13914/16964) | 81.4 (14156/17391) | 83.1 (15223/18310) | 76.6 (14351/18744) | -0.6 | 1.7 | -6.5 |
| Bonthe | 92.9 (7480/8053) | 98.1 (7907/8062) | 107.8 (9152/8488) | 104.7 (9316/8898) | 5.2 | 9.7 | -3.1 |
| Falaba | 111.1 (9147/8236) | 96.5 (8148/8444) | 81.3 (7230/8890) | 76.6 (6969/9100) | -14.6 | -15.2 | -4.7 |
| Kailahun | 87.8 (18530/21112) | 88.0 (19461/22115) | 93.2 (21697/23283) | 94.5 (22032/23327) | 0.2 | 5.2 | 1.3 |
| Kambia | 101.2 (14028/13856) | 94.0 (13305/14156) | 91.4 (13627/14904) | 91.3 (13980/15310) | -7.2 | -2.6 | -0.1 |
| Karene | 88.7 (10153/11453) | 78.5 (9212/11741) | 80.5 (9949/123612) | 77.0 (9745/12654) | -10.2 | 2 | -3.5 |
| Kenema | 96.8 (23688/24462) | 91.9 (22676/24668) | 92.9 (24129/25972) | 89.2 (24127/27027) | -4.9 | 1 | -3.7 |
| Koinadugu | 104.1 (8522/8183) | 102.0 (8553/8389) | 110.1 (9725/8832) | 106.4 (9616/9041) | -2.1 | 8.1 | -3.7 |
| Kono | 83.2 (16880/20299) | 87.7 (17152/19560) | 83.7 (17236/20594) | 76.8 (17217/22428) | 4.5 | -4 | -6.9 |
| Moyamba | 130.0 (16613/12778) | 105.4 (16081/15257) | 100.5 (16147/16063) | 117.2 (16549/14118) | -24.6 | -4.9 | 16.7 |
| Port Loko | 97.3 (20726/21292) | 100.4 (21919/21828) | 100.4 (23063/22981) | 95.8 (22534/23525) | 3.1 | 0 | -4.6 |
| Pujehun | 100.2 (13922/13896) | 108.3 (15431/14243) | 118.2 (17722/14996) | 114.6 (17594/15353) | 8.1 | 9.9 | -3.6 |
| Tonkolili | 103.3 (21292/20615) | 92.1 (20394/22137) | 85.6 (19956/23307) | 83.8 (19093/22777) | -11.2 | -6.5 | -1.8 |
| Western Area Rural | 108.1 (19263/17819) | 107.6 (19806/18401) | 110.7 (21453/19374) | 111.1 (21878/19688) | -0.5 | 3.1 | 0.4 |
| Western Area Urban | 83.1 (35210/42353) | 82.0 (33318/40626) | 89.1 (33110/42772) | 90.7 (42465/46795) | -1.1 | 7.1 | 1.6 |

*Δ%: Delta in coverage rates.
*: Mean annual coverage calculated as the product of dividing the total number of routine immunization doses administered by target population per specific year.
Note: Some coverage rates are >100% because the number of doses administered exceeded the target population.*

**Supplementary Table 6.** **Annual coverage rates of Pentavalent 3 in Sierra Leone’s districts.**

| **Districts** | **Pentavalent 3 Annual Coverage Rate*** | | | | **Δ% 2019-2020** | **Δ%  2021-2020** | **Δ% 2022-2021** |
| --- | --- | --- | --- | --- | --- | --- | --- |
|  | **2019** | **2020** | **2021** | **2022** |  |  |  |
| Bo | 109.2 (25202/23081) | 99.2 (24401/27327) | 96.2 (24902/25893) | 88.6 (22606/25502) | -10 | -3 | -7.6 |
| Bombali | 84.1 (14264/16964) | 82.1 (14284/17391) | 80.8 (14801/18310) | 75.8 (14208/18744) | -2 | -1.3 | -5 |
| Bonthe | 97.1 (7819/8053) | 100.7 (8120/8062) | 113.3  (9618/8488) | 106.8 (9507/8898) | 3.6 | 12.6 | -6.5 |
| Falaba | 110.7 (9115/9152) | 96.4 (8136/8444) | 82.4 (7329/8890) | 77.7 (7075/9100) | -14.3 | -14 | -4.7 |
| Kailahun | 90.4 (19094/21112) | 89.9 (19883/22115) | 93.0 (21660/23283) | 94.7 (22090/23327) | -0.5 | 3.1 | 1.7 |
| Kambia | 102.9 (14261/13856) | 91.7 (12977/14156) | 89.6 (13348/14904) | 89.9 (13766/15210) | -11.2 | -2.1 | 0.3 |
| Karene | 89.0 (10190/11453) | 77.6 (9109/11741) | 80.1 (9900/12362) | 76.3 (9653/12654) | 2.7 | 2.5 | -3.8 |
| Kenema | 95.5 (23360/24462) | 90.7 (22373/24668) | 89.7 (23301/25972) | 88.6 (23958/27027) | -4.8 | -1 | -1.1 |
| Koinadugu | 102 (8343/8183) | 101.1 (8478/8389) | 108.2 (9557/8832) | 107.7 (9741/9041) | -0.9 | 7.1 | -0.5 |
| Kono | 78.8 (15999/20299) | 81.2 (15875/19560) | 78.8 (16219/20594) | 73.0 (16377/22428) | 2.4 | -2.4 | -5.8 |
| Moyamba | 124.9 (15964/12778) | 101.7 (15518/15257) | 97.5 (15656/16064) | 114.3 (16144/14118) | -23.2 | -4.2 | 16.8 |
| Port Loko | 96.2 (20486/21292) | 96.4 (21043/21828) | 98.9 (22745/22981) | 93.0 (21882/23525) | 0.2 | 2.5 | -5.9 |
| Pujehun | 101.2 (14066/13896) | 109.4 (15581/14243) | 111.8 (16761/14996) | 115.6 (17745/15354) | 8.2 | 2.4 | -3.2 |
| Tonkolili | 100.4 (20694/20615) | 86.9 (19246/22137) | 83.4 (19439/23307) | 80.7 (18379/22777) | -13.5 | -3.5 | -2.7 |
| Western Area Rural | 108.0 (19236/17819) | 102.5 (18866/18401) | 110.1 (21330/19374) | 113.1 (22269/19688) | -5.5 | 7.6 | 3 |
| Western Area Urban | 84.0 (35555/42353) | 79.6 (32358/40626) | 90.0 (38498/42772) | 90.6 (42382/46795) | -4.4 | 10.4 | 0.6 |

*Δ%: Delta in coverage rates.
*: Mean annual coverage calculated as the product of dividing the total number of RI doses administered by the target population per specific year.
Note: Some coverage rates are >100% the number of doses administered exceeded the target population.*
